# Current Status and Clinical Characteristics of Familial Hypercholesterolemia Patients in Korea: A Multicenter, Real-World Experience

**DOI:** 10.3390/diagnostics15233062

**Published:** 2025-12-01

**Authors:** Kyung An Kim, Moon-kyung Jung, Eui-Soon Kim, Dongwoo Kim, Joonseok Kim, Hoon Seok Kim, Jong-Chan Youn

**Affiliations:** 1 Division of Cardiology, Department of Internal Medicine, Incheon St. Mary’s Hospital, College of Medicine, The Catholic University of Korea, Seoul 06591, Republic of Korea; 2 Catholic Research Institute for Intractable Cardiovascular Disease, College of Medicine, The Catholic University of Korea, Seoul 06591, Republic of Korea; 3 Division of Cardiology, Department of Internal Medicine, Seoul St. Mary’s Hospital, College of Medicine, The Catholic University of Korea, Seoul 06591, Republic of Korea; dongwoo6249@gmail.com (D.K.); kimjs.ii.mm@gmail.com (J.K.); 4 Department of Laboratory Medicine, Seoul St. Mary’s Hospital, College of Medicine, The Catholic University of Korea, Seoul 06591, Republic of Korea

**Keywords:** familial hypercholesterolemia, genetic testing, lipid-lowering treatment, atherosclerosis

## Abstract

**Background:** Familial hypercholesterolemia (FH) continues to be underrecognized and inadequately treated. We aimed to investigate the current status of FH diagnosis and treatment in South Korea. **Methods:** Patients from two tertiary hospitals in South Korea between 2010 and 2023 with either a diagnosis of FH (ICD-10 code: E7800), had *LDLR*, *APOB*, or *PCSK9* mutations, or had low-density lipoprotein cholesterol (LDL-C) levels exceeding 325 mg/dL were considered for inclusion. Demographic and laboratory characteristics as well as pharmacologic treatment patterns were assessed. **Results:** A total of 148 patients were retrospectively identified. The mean age at diagnosis was 49.3 years, and 33 (22.3%) had a history of established atherosclerotic cardiovascular disease (ASCVD). The majority of patients were diagnosed in the cardiology or endocrinology departments. The LDL-C level at enrollment was 247 ± 98 mg/dL (conversion to treatment-naïve LDL-C: 343 ± 141 mg/dL), which decreased to 122 ± 60 mg/dL after one year, achieving guideline-recommended target levels in 11.5%. A high proportion of patients were treated with statins (80.2%) and ezetimibe (64.9%), but the use of proprotein convertase subtilisin/kexin type 9 inhibitors was low (11.7%). Patients diagnosed after 2020 achieved significantly lower LDL-C levels at one year compared to those diagnosed between 2020 and 2019 (107 ± 50 vs. 152 ± 68 mg/dL, *p* = 0.003). Two ischemic strokes and two myocardial infarctions occurred during a median follow-up of 25.3 months. **Conclusions:** FH is frequently diagnosed late after the onset of clinical ASCVD and is undertreated, although recent trends show improvement. Our results again underline the need for proper screening and identification of patients with FH.

## 1. Introduction

Familial hypercholesterolemia (FH) is an autosomal dominant disorder affecting low-density lipoprotein cholesterol (LDL-C) metabolism, primarily caused by mutations in the *LDLR*, *APOB*, and *PCSK9* genes [1,2]. These genetic alterations result in significantly elevated LDL-C levels, substantially increasing the risk of premature atherosclerotic cardiovascular disease (ASCVD) from childhood [3]. Recent global prevalence estimates for FH range from 1:200 to 1:250, with variations across populations [4,5,6]. A meta-analysis conducted in 2020 estimated the prevalence of FH in Asia at 0.19% compared to 0.32% in Europe and North America [7]. The exact prevalence of FH in Korea is yet to be reported, although it is estimated to be largely similar to other countries [2,7]. However, the prevalence in any given population depends on factors such as the diagnostic criteria used, ethnicity, and the age distribution of the population [8].

Diagnosis of FH is typically based on clinical presentation and is often made through the identification of a positive family history of premature ischemic heart disease and personal hypercholesterolemia [2]. The most widely used diagnostic criteria include the Dutch Lipid Clinic Network (DLCN) and Simon Broome criteria [3,9]. The detection rate for pathologic variants (PVs) in patients diagnosed with FH is 40–80% [10,11,12,13], and the presence of PVs has been shown to correlate with increased risk of cardiovascular disease and poorer response to lipid-lowering therapy [14,15,16]. Despite advancements in FH treatment, a significant proportion of affected individuals remain undiagnosed or inadequately treated [17,18,19]. In Korea, the General Health Screening Program mandates LDL-C testing every 4 years for individuals aged 20 and older, providing a potential platform for identifying high-risk individuals [20]. However, challenges persist in FH detection and management due to factors such as insufficient awareness among healthcare providers and the general population [21]. Therefore, this study aimed to investigate the current status of FH diagnosis and treatment in two large tertiary hospitals in South Korea.

## 2. Methods

### 2.1. Study Design and Study Participants

**Figure 1:**
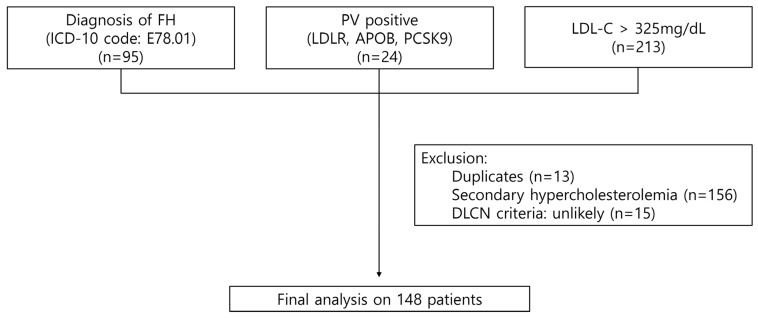
Selection process of the study population. FH, familial hypercholesterolemia; ICD, International Classification of Diseases; LDL-C, low-density lipoprotein cholesterol; DLCN, Dutch Lipid Clinic Network.

We conducted a retrospective analysis of patients from two major tertiary hospitals in Korea between 2010 and 2023. The study included three groups: (1) individuals with a diagnosis of FH based on the ICD-10 code E78.01 (*n* = 95); (2) patients with PVs in the *LDLR*, *APOB*, or *PCSK9* genes (*n* = 24); and (3) individuals with LDL-C levels > 325 mg/dL, corresponding to 8 points on the DLCN criteria and meeting the definition of probable FH based LDL-C levels alone (*n* = 213). After excluding overlapping cases (*n* = 13), patients with secondary hypercholesterolemia (*n* = 156), and those classified as “unlikely” based on the DLCN criteria (*n* = 15), a total of 148 patients were included in the final analysis (Figure 1). The Institutional Review Board of each participating institution including Seoul St. Mary’s Hospital approved this study (KC24RISI0109), and the requirement for informed consent was waived due to its retrospective nature. This study is in accord with the 1975 Declaration of Helsinki.

### 2.2. Definitions

To assess the baseline LDL-C levels of patients who were already on lipid-lowering therapy at the time of diagnosis, we adjusted LDL-C levels to pretreatment levels using the following formula: *Treatment-naïve LDL-C = Current LDL-C/(1 − LDL-C reduction percentage)*, which has been reported to correlate with pretreatment LDL-C levels in FH patients with good accuracy [22]. The LDL-C reduction percentage was based on the known efficacy of the specific lipid-lowering therapy the patient was receiving at the time of measurement, where the reduction percentages for each lipid-lowering medication are listed in Supplementary Table S1 [23,24,25]. Target LDL-C levels were defined as <55 mg/dL in patients with established ASCVD or another major risk factor, and <70 mg/dL in those without these conditions [2].

A patient was considered to have been diagnosed with FH at the time (1) the ICD code E78.01 was assigned, or (2) results for *LDLR*, *APOB*, or *PCSK9* mutations were confirmed. Baseline data including demographics, medical history, and laboratory data were recorded at this time point. For the patients with LDL-C levels > 325 mg/dL, but who had not received an ICD-10 diagnosis of FH or did not have *LDLR*, *APOB*, or *PCSK9* mutations, baseline data were defined as those obtained at the time of the laboratory test that first recorded an LDL-C value > 325 mg/dL. The “diagnosis” was defined as having been made in the department where lipid-lowering therapy was first initiated. Lipid profiles and medical treatment data at one year were obtained from the visit closest to one year after the initial diagnosis. Clinical outcomes, including new myocardial infarction, coronary revascularization, and ischemic stroke, were identified through a review of medical records and followed-up for the maximum available duration. Mortality status was verified based on records of disqualification from the National Health Insurance Service.

### 2.3. Genetic Analysis

Genomic DNA was extracted from peripheral blood samples. The *LDLR* gene was analyzed by direct sequencing, covering all exons and exon–intron boundaries; this testing has been available at our institution since 2019. In addition, we reviewed the records of patients who underwent next-generation sequencing (NGS) between 2019 and 2023 to identify PVs in FH-related genes (*LDLR*, *APOB*, or *PCSK9*), detected either as primary findings or secondary findings from broader testing. NGS was performed using the TruSight One Expanded Sequencing Panel (Illumina, San Diego, CA, USA), which targets approximately 6700 genes associated with Mendelian disorders. Library preparation followed Illumina’s on-bead tagmentation and hybrid-capture enrichment workflow with 50–100 ng of input genomic DNA. Sequencing used 2 × 150 bp paired-end reads on an Illumina NextSeq platform, and data were processed using standard enrichment-based pipelines, including alignment to the GRCh37/hg19 reference genome and variant calling with Illumina’s recommended analysis suite. Variants were subsequently annotated and reviewed using VariantStudio (Illumina, San Diego, CA, USA). The pathogenicity of all identified variants was determined according to the 2015 American College of Medical Genetics and Genomics and the Association for Molecular Pathology guidelines [26]. All PVs are reported using standard Human Genome Variation Society nomenclature. In the primary analysis, only patients with a positive PV result were included. However, baseline characteristics were also compared between PV-positive and PV-negative patients among all individuals who underwent PV testing.

### 2.4. Statistical Analysis

Categorical data are presented as numbers and frequencies and compared using the χ2 test, or Fisher’s exact test when more than 20% of cells had an expected count of less than 5 in a 2 × 2 table. Continuous variables are expressed as the mean ± standard deviation or median [interquartile range], depending on variable distribution, and compared using the Student’s t-test or Mann–Whitney U test as appropriate. Data on family history were not systematically recorded and contained a high proportion of missing values; for statistical analysis, these missing entries were considered as negative findings. Use of lipid-lowering medications and corresponding changes in lipid profiles were analyzed among patients with available one-year follow-up data. To analyze temporal trends in FH treatment, patients were stratified according to the year of diagnosis: 2010–2019 and 2020–2023, to reflect changes in lipid-lowering management following the introduction of lower LDL-C targets in the 2019 ESC/EAS guidelines and the initiation of national insurance coverage for Proprotein Convertase Subtilisin/Kexin Type 9 (PCSK9) inhibitors in 2020. Changes in LDL-C over time were analyzed using a repeated-measures linear mixed-effects model including time (baseline and 1-year), diagnosis period (2010–2019 vs. 2020–2023), and their interaction as fixed effects, with a random intercept for each participant. The model was adjusted for age, sex, and ASCVD history to assess whether LDL-cholesterol reduction differed between diagnosis periods after accounting for covariates. A two-sided *p*-value of less than 0.05 was considered statistically significant. Statistical analyses were performed using R Statistical Software version 4.4.2 (R Foundation for Statistical Computing, Vienna, Austria).

## 3. Results

### 3.1. Baseline Characteristics

**Figure 2:**
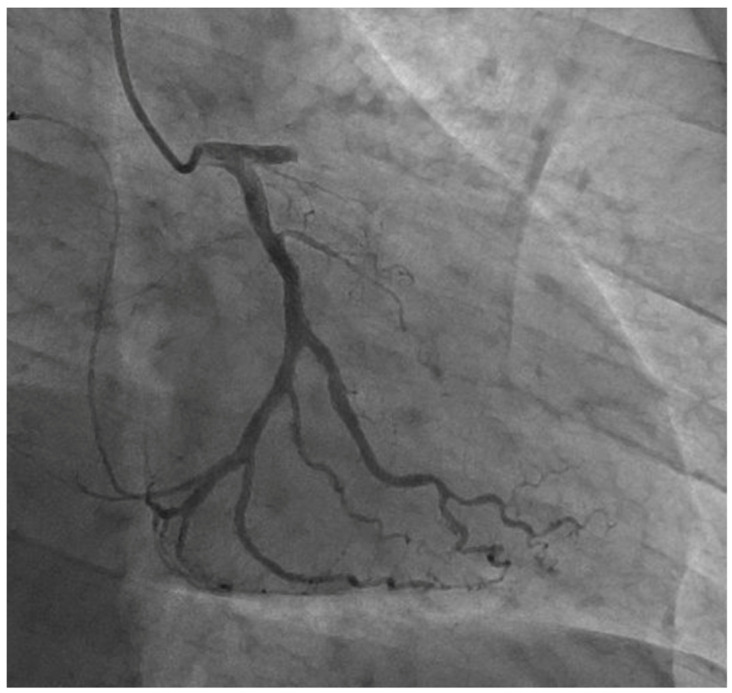
Representative coronary angiogram of a 42-year-old male with ST-segment elevation myocardial infarction.

The baseline characteristics of our study subjects are presented in Table 1. The mean age at diagnosis was 49.3 years, and 60.8% were female. There were 9 (6.1%) patients with a history of previous myocardial infarction, 16 (10.8%) patients with a history of percutaneous coronary intervention, and 5 (3.4%) patients with a history of stroke. The initial LDL-C levels averaged 242.0 ± 97.9 mg/dL, which, when converted to treatment-naïve LDL-C levels, corresponded to 343.3 ± 140.9 mg/dL. When we categorized our study subjects according to the DLCN criteria, possible FH (score 3–5), probable FH (score 6–8), and definite FH (score > 8) were found in 27 (18.2%), 57 (38.5%), and 64 (43.2%), respectively. A representative coronary angiogram of a 42-year-old male with ST-segment elevation myocardial infarction is shown in Figure 2.

### 3.2. Clinical Presentation and Reasons for Referral

We investigated the pathways leading to the diagnosis of FH. The most common referral reason was marked dyslipidemia, observed in 55 (37.2%) patients, of whom 30 (20.3%) were referred without prior lipid-lowering therapy and 25 (16.9%) after persistently elevated LDL-C levels despite treatment. Coronary artery disease (CAD) accounted for 26 (17.6%) cases—5 (3.4%) with asymptomatic CAD, 17 (11.5%) with angina, and 4 (2.7%) with myocardial infarction—in which persistently high LDL-C levels despite lipid-lowering therapy prompted further evaluation for FH. Seven (4.7%) patients were diagnosed after embolic events, such as stroke or retinal artery occlusion. Referrals based on xanthomas and family history were less common, with 2 (1.1%) patients and 3 (2.0%) patients, respectively. Additionally, 48 (32.4%) patients were referred after elevated LDL-C levels were incidentally detected during evaluations for other conditions, such as arrhythmias, diabetes, hypertension, or pre-operative assessments. Of note, 7 (4.7%) patients with elevated LDL-C levels > 325 mg/dL did not receive lipid-lowering treatment and were presumably undiagnosed (Table 2).

**Figure 3:**
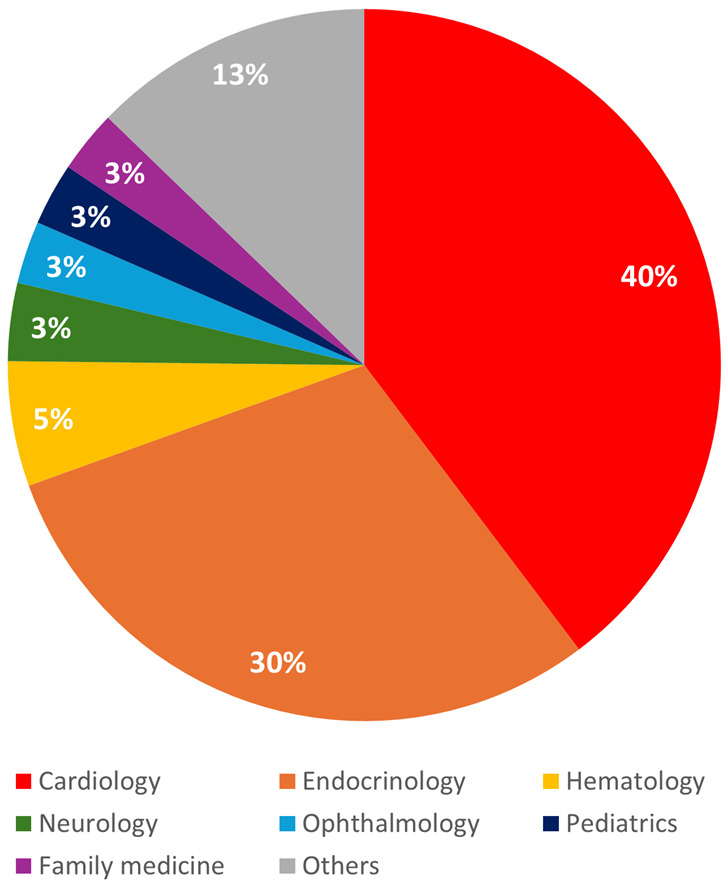
Distribution of familial hypercholesterolemia diagnoses by clinical department.

Among the medical specialties involved in the diagnosis and treatment of FH, cardiology accounted for the largest proportion at 40%, followed by endocrinology at 30% (Figure 3). Neurology accounted for a relatively smaller proportion at 3%. FH was also diagnosed in various specialties including hematology and ophthalmology when elevated LDL-C levels were identified as an incidental finding during evaluations for other medical conditions.

### 3.3. Pathologic Variant Testing

Out of 148 participants, 47 underwent genetic testing. Among them, 45 individuals from the cardiology and endocrinology departments underwent *LDLR* gene testing due to elevated LDL-C levels, with 22 identified as PV-positive. The list of PVs are shown in Table 3. A broad spectrum of pathogenic or likely pathogenic *LDLR* variants was identified, encompassing missense, nonsense, frameshift, splice-site, and indel mutations. Among these, three recurrent variants were observed: c.361T>G [p.Cys121Gly] in five patients, c.2054C>T [p.Pro685Leu] in two patients, and c.682G>T [p.Glu228]* in two patients. In addition, NGS identified PVs in the *LDLR* gene in two pediatric patients who underwent testing for retinal disorders, where these findings were incidental and unrelated to the primary clinical indication. One of these patients also carried a PV in *APOB*, and no PVs were detected in *PCSK9*.

In a supplementary analysis of all patients who underwent *LDLR* gene testing, out of a total of 73 individuals who were tested, 24 (33.9%) were positive, and 49 (67.1%) were negative (Supplementary Table S2). The PV-positive patients were slightly younger (mean age 55.2 vs. 47.5 years) and had slightly higher LDL-C levels, although this did not reach statistical significance. PV-positive patients also had lower blood pressures, lower triglycerides, and lower platelet count, and were more likely to be prescribed PCSK9 inhibitors.

### 3.4. Lipid Profiles and Lipid Lowering Treatments

Follow-up data on lipid profiles and lipid-lowering treatments up to one year were available for 96 patients (Table 4). After one year of treatment, the mean LDL-C decreased to 121.6.1 ± 60.0 mg/dL, achieving target levels in 11 (11.5%) patients. Lipid-lowering therapies at the one-year point included statin therapy for 77 (80.2%) patients, ezetimibe for 61 (64.9%) patients, fibrates for 7 (7.4%) patients, and Proprotein convertase subtilisin/kexin type 9 (PCSK9) inhibitors for 11 (11.7%) patients.

**Figure 4:**
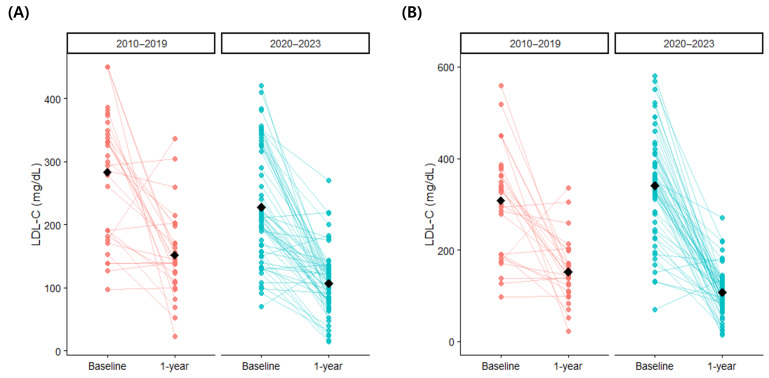
One-year changes in low-density lipoprotein cholesterol according to diagnosis period: (**A**) uncorrected values and (**B**) converted treatment-naïve baseline values. LDL-C, low-density lipoprotein cholesterol.

When the patients were stratified according to the year of diagnosis, 33 (34.4%) were diagnosed between 2010 and 2019, and 63 (65.6%) were diagnosed in 2020 or later. Those diagnosed from 2020 onwards had lower baseline LDL-C levels, but there was no significant difference after conversion to treatment-naïve LDL-C levels (Figure 4, Table 4). Patients diagnosed from 2020 onwards were more likely to be receiving statins, higher statin doses, and ezetimibe. At one year after follow-up, the patients diagnosed from 2020 onwards had significantly lower LDL-C (2010–2019: 152.1 ± 68.3 mg/dL vs. 2020–2023: 106.8 ± 49.8 mg/dL, *p* = 0.003), total cholesterol (*p* < 0.001), and triglycerides (*p* = 0.021) compared to those diagnosed between 2010 and 2019. Achievement of guideline-recommended target LDL-C levels were low in both groups. The proportion of patients on statins and statin doses were similar between the two groups, but those diagnosed from 2020 onwards were more likely to receive ezetimibe (2010–2019: 43.8% vs. 2020–2023: 75.8%, *p* = 0.004) and PCSK inhibitors (2010–2019: 0.0% vs. 2020–2023: 17.7%, *p* = 0.028), and less likely to receive fibrates (*p* = 0.010).

Analysis on LDL-C levels across the period of diagnosis using linear mixed-effects models showed that there was a significant interaction between the degree of LDL-C reduction and the period of diagnosis when the baseline LDL-C was estimated using conversion to treatment-naïve levels (Table 5). The magnitude of LDL-C reduction from treatment-naïve baseline levels for patients diagnosed in 2020 or later was 73.2 mg/dL (95% confidence interval, 12.2–134.8 mg/dL; *p* = 0.026) greater than those diagnosed between 2010 and 2019, even after adjustment for overall LDL-C levels, age, sex, and prior ASCVD. When unadjusted LDL-C levels were used for baseline values, no difference in 1-year LDL-C reduction was noted across diagnosis periods, but overall LDL-C levels were significantly lower in the patients diagnosed in 2020 or later (*p* = 0.002).

### 3.5. Clinical Outcomes

During a median follow-up period of 25.3 months, the clinical outcomes included two cases of ischemic stroke and two cases of myocardial infarction. No mortality was reported.

## 4. Discussion

This study retrospectively identified patients with FH through ICD-10 code diagnosis, genetic testing, and elevated LDL-C levels. The main findings are as follows: (1) a substantial proportion of patients with suspected FH are diagnosed later in life, often after the cumulative effects of prolonged LDL-C elevation have manifested; (2) many patients are incidentally diagnosed during evaluation for unrelated medical conditions; and (3) despite lipid-lowering therapy, attainment of guideline-recommended LDL-C targets remains suboptimal. Our study, in line with previous research, provides evidence of the persistent insufficient awareness of FH, resulting in significant underdiagnosis and undertreatment [17,18,19].

There is no universally accepted diagnostic standard for FH, and different clinical criteria are applied across countries [27]. The most commonly used diagnostic criteria worldwide are the DLCN and the Simon Broome criteria [3,9]. Both incorporate LDL-C levels as a key component of the diagnostic algorithm, as well as physical examination findings, patient and family history, and the presence of PVs. Of note, these criteria were largely derived and validated in Western populations. In an analysis of the LDL-C distribution of FH patients from the 2020 Korean Familial Hypercholesterolemia (KFH) Registry, an LDL-C level of 177 mg/dL was suggested as a threshold for suspecting FH, while the median LDL-C level was 221 mg/dL [10]. The LDL-C levels in the present study were somewhat higher, with a mean of 242 mg/dL and an estimated treatment-naïve level of 327 mg/dL. This may have been in part due to the selection criteria of LDL-C > 325 mg/dL, corresponding to a diagnosis of probable FH on LDL-C levels alone. Consistent with this, our cohort included a higher proportion of patients with definite or probable FH compared with the 2020 KFH registry, suggesting that our study population may represent a cohort with greater phenotypic specificity for FH.

The most common reason for referral was dyslipidemia, followed by manifestations of ASCVD such as CAD, stroke, and retinal artery occlusion. Nearly half of the cases were identified in cardiology, often after initiation of lipid-lowering therapy for established CAD. Early screening beginning in childhood, combined with cascade testing, has been implemented in several European countries to facilitate timely identification and treatment of FH [28]. In Korea, integration of a targeted screening approach of those with high LDL-C levels through the General Health Screening Program has shown efficacy for the detection of FH in the general population [20]. In our study, the high proportion of FH diagnoses made only after the onset of clinical ASCVD indicates delayed detection, further supporting the need for earlier diagnosis such as through national screening initiatives. In addition, a substantial proportion of FH diagnoses occurred incidentally when elevated LDL-C levels were detected during evaluations for unrelated conditions—including arrhythmias, diabetes, hypertension, malignancy work-ups, routine health examinations, and pre-operative assessments. These incidental cases often had LDL-C levels far exceeding guideline thresholds and frequently met the DLCN criteria once evaluated, suggesting delayed recognition rather than milder phenotypes. These findings highlight gaps in Korea’s FH screening infrastructure and underscore the need for more proactive detection strategies to enable earlier intervention.

Accurate identification of PVs and their clinical interpretation is important in FH management, especially as those with PVs have markedly higher cardiovascular risk even after accounting for LDL-C levels [14,29]. However, there is no official national guideline for genetic testing in FH screening, and insurance coverage criteria for genetic testing are limited in Korea. Currently, it is performed in cases where FH is strongly suspected clinically, in individuals with a family history of early onset CVD, in first-degree relatives of diagnosed FH patients, and in patients with severe high LDL-C who show an insufficient response to lipid-lowering therapy [2,20]. In our study, only 31.8% underwent PV testing, which is likely due to barriers such as cost and insufficient awareness, and reflects the current challenges of FH treatment in Korea. In our study, positive findings were found in 34% of the patients tested for PVs, similar to the 35% found in the 2020 Korean Registry Project [10], but further large-scale studies will be needed to accurately estimate the prevalence of PVs in Korea, which is known to vary significantly across ethnicities [30,31,32]. Several of the recurrent variants in our cohort have been reported as pathogenic in multiple FH families and are listed among recurrent LDLR mutations in East Asian and global FH variant compilations [33]. However, most of the remaining variants in our series were observed only once or twice and were not among the pan-Asian founder mutations, consistent with the extensive allelic heterogeneity described in FH [34].

In addition, two pediatric patients were incidentally found to harbor PVs in the *LDLR* and/or *APOB* genes through clinical exome sequencing initially performed for retinal disorders. Such findings illustrate that when genetic testing involves broad sequencing approaches, such as whole-exome or whole-genome sequencing, clinically relevant variants unrelated to the primary indication may be detected. However, the practice of using wide genomic coverage for the purpose of identifying incidental findings remains controversial in clinical genetics, given the potential for numerous secondary findings and the complexities they raise for patient counseling and management [35].

Treatment for FH typically follows a stepwise approach, starting with high-intensity statins at maximum tolerated doses, followed by the addition of ezetimibe [2,3]. If this fails to achieve target LDL-C levels, PCSK9 inhibitors should be considered [2,3]. As patients with FH have increased ASCVD risk beyond LDL-C levels, stringent lipid level control is key to preventing ASCVD [14]. The optimal treatment targets for FH patients are a 50% reduction in LDL-C and levels < 70 mg/dL, or <55 mg/dL in the presence of ASCVD [2,3], but achievement of these targets is difficult in the majority of patients [18]. LDL-C < 70 mg/dL in patients with established ASCVD or major risk factors and <100 mg/dL in those without these conditions are recommended as more realistic targets in many guidelines [2,36]. Our findings support the efficacy of contemporary lipid-lowering therapy, demonstrating a reduction in mean LDL-C levels from 247 mg/dL to 122 mg/dL after one year of treatment. However, achievement of target LDL-C levels was limited to only 11.5% of the population. Although this is comparable to global data showing that only 2.8% and 13.6% of FH patients achieve an LDL-C level of <70 mg/dL and <100 mg/dL, respectively, substantial room for improvement remains [18]. Previous studies have shown limited probability of achieving targets even with maximal statin-based lipid-lowering therapies [37,38,39]. Recent advances in lipid-lowering therapy, including PCSK9 inhibitors and bempedoic acid, have demonstrated efficacy in patients who fail to achieve target LDL-C levels or are intolerant to statin-based regimens, and may offer additional therapeutic options for those with FH [40,41]. The low proportion of patients receiving PCSK9 inhibitors (11.7%) at one year despite limited LDL-C target attainment reflects persistent barriers at both the system and clinician levels. Early in the study period, PCSK9 inhibitors were not widely accessible, and national insurance reimbursement for FH and very high-risk ASCVD patients only began in 2020, markedly limiting uptake among those diagnosed earlier. Even after reimbursement, out-of-pocket costs likely remained a deterrent. Institutional prescribing patterns, availability constraints, and clinical inertia may also have contributed, particularly when existing therapy was perceived as sufficient despite suboptimal control. Because PCSK9 inhibitors provide substantial additional LDL-C lowering in patients with suboptimal response to statin-based regimens [39,42], reducing financial and administrative obstacles will be important to improve long-term lipid management and cardiovascular outcomes in this population.

In our study, patients diagnosed from 2020 onward had significantly lower LDL-C levels at baseline and at one year. The absolute magnitude of LDL-C reduction from baseline to one year was similar between diagnosis periods; however, when baseline LDL-C was estimated using conversion to treatment-naïve levels, patients diagnosed from 2020 onward showed significantly greater LDL-C reduction. This enhanced response likely reflects a broader use of ezetimibe and the increased availability and reimbursement of PCSK9 inhibitors beginning in 2020, consistent with the significantly higher use of these therapies in patients diagnosed during this later period. In addition, evolving clinical practice influenced by the publication of guidelines recommending lower LDL-C targets, the introduction of PV testing for more definite FH diagnoses (which was available in our institutions from 2019), and heightened clinician awareness may have contributed to earlier recognition and more intensive therapy. As seen by the higher use of PCSK9 in PV positive patients, limited genotyping may obscure true genotype–phenotype relationships and hinder optimal risk stratification and treatment planning, highlighting the need for systematic PV testing. Early and intensive lipid-lowering is known to reduce cardiovascular risk in FH, consistent with the low number of cardiovascular events observed during follow-up in our cohort. Collectively, these findings underscore the importance of active screening for early FH diagnosis and aggressive lipid-lowering treatment to improve long-term cardiovascular outcomes.

## 5. Limitations

Our study has several limitations that should be acknowledged. First, this study was conducted at two tertiary hospitals with a relatively small cohort size. As such hospitals often manage more severe or well-resourced patients, the cohort may not fully reflect the general FH population, particularly given that diagnoses were made in the cardiology department after clinical ASCVD. Second, some patients with an ICD-10 code diagnosis of FH may have been misdiagnosed. In addition, the different methods used to identify patients with FH may have resulted in heterogeneity of the study cohort. However, we applied the DLCN criteria and excluded those who did not meet diagnostic criteria. Third, due to the retrospective nature of the study, we were unable to comprehensively assess important patient characteristics, including xanthomas, medical and family histories, and laboratory data such as lipoprotein(a). Finally, the rate of adverse events in the study population was low, limiting the ability to analyze outcome data.

## 6. Conclusions

In this real-world analysis of patients with FH, many were diagnosed later in life and initiated treatment only after the onset of clinical ASCVD. Although LDL-C levels decreased substantially and cardiovascular events were infrequent during lipid-lowering therapy, attainment of target LDL-C levels was low. Our study underscores the persistent challenges in the diagnosis and management of FH, highlighting its underdiagnosis and undertreatment. However, comparison across diagnosis periods suggests improvement in the treatment of FH, as evidenced by lower LDL-C levels in recent years. Active screening for early diagnosis of FH and intensive lipid-lowering treatment will be crucial in addressing the cardiovascular burden associated with FH and enhancing long-term outcomes for affected individuals.

## Figures and Tables

**Table 1:** Baseline characteristics of the study population.

Characteristics	Entire Population (*n* = 148)
Demographics and medical history	
Age (years)	49.3 ± 17.2
Sex	
Male (%)	58 (39.2)
Female (%)	90 (60.8)
BMI (kg/m^2^)	23.7 ± 3.9
History of smoking	
Non-smoker	105 (70.9)
Ex-smoker	31 (20.9)
Current smoker	12 (8.1)
Diabetes (%)	20 (13.5)
Hypertension (%)	34 (23.0)
Previous MI (%)	9 (6.1)
Previous PCI (%)	16 (10.8)
Previous Stroke (%)	5 (3.4)
Family History	
MI in 1st degree relative (%)	16 (10.8)
MI in 2nd degree relative (%)	11 (7.4)
Xanthoma in 1st degree relative (%)	2 (1.4)
Xanthoma in 2nd degree relative (%)	2 (1.4)
Dyslipidemia in 1st degree relative (%)	37 (25.0)
Dyslipidemia in 2nd degree relative (%)	13 (8.8)
Clinical and Laboratory Characteristics	
Systolic blood pressure (mmHg)	126.0 ± 18.5
Diastolic blood pressure (mmHg)	78.2 ± 13.7
Total cholesterol (mg/dL)	337.6 ± 130.3
HDL-cholesterol (mg/dL)	54.6 ± 17.7
LDL-cholesterol (mg/dL)	242.0 ± 97.9
Conversion to treatment-naïve (mg/dL)	343.3 ± 140.9
Triglycerides (mg/dL)	192.3 ± 161.5
Hemoglobin (mg/dL)	13.9 ± 1.9
WBC count (10^9^/L)	6.7 ± 2.3
Platelet count (10^9^/L)	248.6 ± 80.1
hs-CRP (mg/dL)	0.4 ± 1.0
Glucose (mg/dL)	108.3 ± 42.8
HbA1c (%)	6.1 ± 1.4
eGFR (mL/min/1.73m^2^)	94.7 ± 24.9
Uric acid (mg/dL)	5.5 ± 4.8
Medical treatment	
Glucose-lowering medication (%)	16 (10.8)
Antihypertensive medication (%)	39 (26.3)
Lipid-lowering medication (%)	71 (48.0)
Antiplatelet (%)	18 (12.2)
Diagnosis	
PV tested	47 (31.8)
PV-positive	24 (16.2)
DLCN criteria	
Definite (%)	64 (43.2)
Probable (%)	57 (38.5)
Possible (%)	27 (18.2)
ICD diagnosis of FH (%)	61 (41.2)

PV, pathologic variant; BMI, body-mass index; MI, myocardial infarction; PCI, percutaneous coronary intervention; HDL, high-density lipoprotein; LDL, low-density lipoprotein; WBC, white blood cell, hs-CRP, high-sensitivity C-reactive protein; HbA1c, glycated hemoglobin; eGFR, estimated glomerular filtration rate; DLCN, Dutch Lipid Clinic Network; ICD, International Classification of Diseases.

**Table 2:** Clinical presentation leading to evaluation and diagnosis of familial hypercholesterolemia.

	*n* (%)
1. CAD	26 (17.6)
(1) Asymptomatic CAD	5 (3.4)
(2) Angina or equivalent	17 (11.5)
(3) Myocardial infarction	4 (2.7)
2. Embolic event	7 (4.7)
(1) Stroke	5 (3.4)
(2) Retinal artery occlusion	2 (1.4)
3. Dyslipidemia	55 (37.2)
(1) Without previous lipid-lowering treatment	30 (20.3)
(2) With previous lipid-lowering treatment	25 (16.9)
4. Xanthoma	2 (1.1)
5. Family History	3 (2.0)
6. During in-hospital evaluation for other medical conditions	48 (32.4)
(1) Arrhythmia	6 (4.1)
(2) Hypertension	1 (0.7)
(3) Diabetes	3 (2.0)
(4) Malignancy	7 (4.7)
(5) Health examination	6 (4.1)
(6) Genetic testing	2 (1.4)
(7) Pre-operative evaluation	4 (2.7)
(8) Other	19 (12.8)
7. Undiagnosed	7 (4.7)

CAD, coronary artery disease.

**Table 3:** Pathologic variant list.

	c.DNA Change	Protein Change	Type
1	LDLR c.2054C>T	p.Pro685Leu	Pathogenic
2	LDLR c.361T>G	p.Cys121Gly	Pathogenic
3	LDLR c.361T>G	p.Cys121Gly	Likely pathogenic
4	LDLR c.1448G>A	p.Trp483*	Pathogenic
5	LDLR c.682G>T	p.Glu228*	Pathogenic
6	LDLR c.313 + G>A	splicing error	Pathogenic
7	LDLR c.361T>G	p.Cys121Gly	Likely pathogenic
8	LDLR c.361T>G	p.Cys121Gly	Likely pathogenic
9	LDLR c.301G>A	p.Glu101Lys	Pathogenic
10	LDLR c.395G>A	p.Arg132Gln	VUS
11	LDLR c.1195G>A	p.Ala339Thr	Likely pathogenic
12	LDLR c.661G>A APOB c.1599_1611del	p.Asp221Asn p.Met534ThrfsTer28	Pathogenic Likely pathogenic
13	LDLR c.280G>A	p.Asp94Asn	Likely pathogenic
14	LDLR c.661G>A	p.Asp221Asn	Pathogenic
15	LDLR c.241C>A	p.Arg81Ser	Likely pathogenic
16	LDLR c.337G>T	p.Glu113Ter	Pathogenic
17	LDLR c.280G>A LDLR c.352G>A	p.Asp94Asn p.Asp118Asn	Likely pathogenic VUS
18	LDLR c.418G>A LDLR c.2333G>T	p.Glu140Lys p.Arg778Ile	Pathogenic VUS
19	LDLR c.2416dup	p.Val806Glyfs*11	Pathogenic
20	LDLR c.621C>T	p.Gly207*	Likely pathogenic
21	LDLR c.361T>G	p.Cys121Gly	Pathogenic
22	LDLR c.2054C>T	p.Pro685Leu	Pathogenic
23	LDLR c.-84G>A	?	VUS
24	LDLR c.682G>T	p.Glu228*	Pathogenic

**Table 4:** Lipid-lowering treatments and lipid profiles in patients with follow-up data.

Characteristics	Entire Population (*n* = 96)	According to Period of Diagnosis		
		Diagnosed 2010-2019 (*n* = 33)	Diagnosed 2020-2023 (*n* = 63)	*p*-Value
Initial visit				
Total cholesterol (mg/dL)	349.1 ± 139.7	408.7 ± 190.7	318.8 ± 92.9	0.028
HDL-cholesterol (mg/dL)	53.9 ± 16.6	55.0 ± 17.7	53.4 ± 16.1	0.379
LDL-cholesterol (mg/dL)	246.6 ± 96.4	283.2 ± 102.4	228.0 ± 88.4	0.008
Conversion to treatment-naïve (mg/dL)	347.1 ± 133.9	328.9 ± 139.4	356.3 ± 131.2	0.348
Triglycerides (mg/dL)	210.9 ± 136.7	336.1 ± 224.5	147.3 ± 120.4	0.065
Statin (%)	43 (44.8)	5 (18.2)	38 (61.3)	<0.001
Dose (mg) ^a^	20 [0, 40]	10 [0, 20]	20 [5, 40]	0.002
High-intensity (%) ^b^	35 (58.3)	4 (36.4)	31 (63.3)	0.195
Ezetimibe (%)	29 (30.2)	4 (12.1)	25 (39.7)	0.010
Fibrate (%)	3 (3.2)	2 (6.1)	1 (1.6)	0.271
PCSK9 inhibitor (%)	3 (3.2)	0 (0.0)	3 (4.8)	0.549
Followed up at 1 year				
Total cholesterol (mg/dL)	208.4 ± 65.4	246.8 ± 69.6	188.6 ± 53.8	<0.001
HDL-cholesterol (mg/dL)	56.4 ± 12.9	57.4 ± 14.8	56.0 ± 12.1	0.662
LDL-cholesterol (mg/dL)	121.6 ± 60.0	152.1 ± 68.3	106.8 ± 49.8	0.003
Change from initial visit (mg/dL)	−119.4 ± 101.1	−118.4 ± 127.0	−119.9 ± 87.7	0.955
Change from treatment-naïve level (mg/dL)	−222.3 ± 153.7	−161.4 ± 163.3	−250.7 ± 141.7	0.010
Target level achievement (%) ^c^	11 (11.5)	2 (6.9)	9 (15.0)	0.321
Triglycerides (mg/dL)	129.6 ± 94.2	170.5 ± 132.8	109.2 ± 58.6	0.021
Statin (%)	77 (80.2)	26 (77.4)	51 (81.6)	0.800
Dose (mg) ^a^	40 [10, 80]	20 [5, 40]	40 [10, 80]	0.143
High-intensity (%)	50 (54.3)	14 (45.2)	36 (59.0)	0.298
Ezetimibe (%)	61 (64.9)	14 (43.8)	47 (75.8)	0.004
Fibrate (%)	7 (7.4)	6 (18.8)	1 (1.6)	0.010
PCSK9 inhibitor (%)	11 (11.7)	0 (0.0)	11 (17.7)	0.028

^a^. Converted to equivalent dose of atorvastatin. ^b^. Equivalent dose of atorvastatin 40 mg or higher. ^c^. Target LDL-cholesterol levels as recommended by the 2022 Consensus statement on the management of familial hypercholesterolemia in Korea. HDL, high-density lipoprotein; LDL, low-density lipoprotein.

**Table 5:** Changes in low-density lipoprotein cholesterol between baseline and at one year analyzed using linear mixed-effects models.

Covariates	Uncorrected Baseline LDL-C		Treatment-Naïve Baseline LDL-C	
	β Coefficient (95% CI)	*p*-Value	β Coefficient (95% CI)	*p*-Value
Overall LDL-C reduction after 1 year	−130.6 (−165.5, −94.6)	<0.001	−339.7 (−408.3, −279.2)	<0.001
Overall LDL-C levels according to periods		0.002		0.401
2010–2019	reference		reference	
2020–2023	−53.7 (−90.6, −23.4)		19.8 (−29.9, 65.1)	
Difference in 1-year LDL-C reduction between periods		0.688		0.026
2010–2019	reference		reference	
2020–2023	9.5 (−35.0, 55.9)		−73.2 (−134.8, −12.2)	
Age (per 1 year)	−0.73 (−1.60, 0.03)	0.075	−0.33 (−1.50, −0.62)	0.532
Male sex	7.0 (−16.3, 28.1)	0.562	21.6 (−11.4, 58.3)	0.173
Prior ASCVD	−35.1 (−73.6, 5.5)	0.103	20.6 (−32.1, 78.0)	0.465

LDL-C, low-density lipoprotein cholesterol; CI, confidence interval; ASCVD, atherosclerotic cardiovascular disease.

## Data Availability

The data supporting this study can be obtained from the corresponding author upon reasonable request. Access will be provided in minimally anonymized form as required by IRB regulations.

## Supplementary Materials

The following supporting information can be downloaded at: https://www.mdpi.com/article/10.3390/diagnostics15233062/s1, Table S1: Low-density lipoprotein cholesterol reduction percentages for each lipid-lowering medication for use in conversion to treatment-naïve low-density lipoprotein cholesterol levels; Table S2: Baseline characteristics of the patients who underwent testing for pathologic variants.
