# Supplementary material for: Current Status and Clinical Characteristics of Familial Hypercholesterolemia Patients in Korea: A Multicenter, Real-World Experience
Source: Diagnostics (Basel). 2025 Dec 1;15(23):3062. doi: 10.3390/diagnostics15233062 (PMC12691502; doi:10.3390/diagnostics15233062)
Supplement: Supplementary file 1 [file diagnostics-15-03062-s001.zip › diagnostics-3994682-supplementary.pdf]

Supplementary Material

Supplementary Table S1. Low-density lipoprotein cholesterol reduction percentages for each lipid-lowering medication for use in conversion to treatment-naïve low-density lipoprotein cholesterol levels

	Reduction in LDL-C from baseline				
	23% [a]	34%	41%	48%	55%
atorvastatin		10mg	20mg	40mg	80mg
rosuvastatin			5mg	10mg	20mg
simvastatin		20mg	40mg	80mg	
pravastatin		40mg	80mg		
pitavastatin		2mg	4mg		
ezetimibe [a]	10mg				

a. The low-density lipoprotein lowering effect of ezetimibe was based on the efficacy of ezetimibe used in combination with statins, since all patients who received ezetimibe were also on statins. LDL-C, low-density lipoprotein cholesterol.

Supplementary Table S2. Baseline characteristics of the patients who underwent testing for pathologic variants

Characteristics	PV-negative (n=49)	PV-positive (n=24)	p-value
Demographics and medical history			
Age (years)	55.2±15.2	47.5±20.3	0.07
Sex			0.41
Male (%)	29 (59.2)	11 (45.8)	
Female (%)	20 (40.8)	13 (54.2)	
BMI (kg/m^2)	24.5±3.1	24.0±4.2	0.59
History of smoking			0.41
Non-smoker	25 (59.5)	16 (76.2)	
Ex-smoker	9 (21.4)	3 (14.3)	
Current smoker	8 (19.0)	2 (9.5)	
Diabetes (%)	6 (12.5)	5 (21.7)	0.51
Hypertension (%)	19 (38.8)	10 (43.5)	0.90
Previous MI (%)	7 (14.6)	5 (21.7)	0.68
Previous PCI (%)	18 (37.5)	9 (39.1)	0.99
Previous Stroke (%)	4 (8.3)	1 (4.3)	0.91
Family History			
MI in 1st degree relative (%)	6 (17.1)	3 (15.8)	1.00
MI in 2nd degree relative (%)	3 (9.4)	4 (22.2)	0.41
Xanthoma in 1st degree relative (%)	0 (0.0)	1 (5.9)	0.80
Xanthoma in 2nd degree relative (%)	0 (0.0)	1 (5.9)	0.80
Dyslipidemia in 1st degree relative (%)	10 (33.3)	5 (27.8)	0.94
Dyslipidemia in 2nd degree relative (%)	1 (3.6)	0 (0.0)	1.00
Laboratory Characteristics			
Systolic blood pressure (mmHg)	132.4±17.3	124.9±15.7	0.09
Diastolic blood pressure (mmHg)	84.0±12.9	73.2±13.8	<0.01
Total cholesterol (mg/dL)	254.4±72.1	260.9±92.8	0.76
HDL-cholesterol (mg/dL)	54.0±12.9	51.5±10.5	0.44
LDL-cholesterol (mg/dL)	161.6±67.1	190.3±95.3	0.21
conversion to treatment-naïve (mg/dL)	325.0±113.4	385.2±189.4	0.18
Triglycerides (mg/dL)	167.6±127.9	112.0±75.6	0.03
Hemoglobin (mg/dL)	14.1±2.0	14.3±1.5	0.75
WBC count (10^9/L)	6.9±2.2	7.6±2.7	0.29
Platelet count (10^9/L)	273.5±61.6	228.5±55.2	0.01

hs-CRP (mg/dL)	0.4±1.3	0.2±0.3	0.35
Glucose (mg/dL)	106.2±22.4	103.4±13.9	0.53
HbA1c (%)	6.0±1.1	5.8±0.5	0.35
Blood urea nitrogen (mg/dL)	15.1±4.4	14.3±4.5	0.48
Creatinine (mg/dL)	0.9±0.2	0.8±0.3	0.64
eGFR (mL/min/1.73m^2)	87.2±18.3	91.3±21.3	0.41
Uric acid (mg/dL)	5.1±1.4	4.9±1.1	0.65
Medical treatment			
Glucose-lowering medication (%)	3 (6.1)	5 (20.8)	0.14
Antihypertensive medication (%)	19 (38.8)	10 (41.7)	0.99
Lipid-lowering medication (%)	45 (91.8)	20 (90.9)	1.00
Antiplatelet (%)	17 (34.7)	12 (50.0)	0.32
Diagnosis			
DLCN criteria			<0.001
Definite (%)	18 (36.7)	22 (91.7)	
Probable (%)	13 (26.5)	2 (8.3)	
Possible (%)	18 (36.7)	0 (0.0)	
ICD diagnosis of FH (%)	2 (4.1)	9 (37.5)	<0.01
Followed up at one year			
	(n=31)	(n=14)	
Total cholesterol (mg/dL)	167.3±21.1	165.6±55.7	0.92
HDL-cholesterol (mg/dL)	51.1±11.7	52.4±10.4	0.72
LDL-cholesterol (mg/dL)	90.2±25.1	89.4±53.5	0.96
Triglycerides (mg/dL)	143.5±117.8	100.1±65.8	0.12
Statin (%)	31 (96.9)	14 (100.0)	0.56
Statin dose (mg) [a]	45.9±25.9	56.4±25.9	0.21
Ezetimibe (%)	26 (81.2)	14 (100.0)	0.21
Fibrate (%)	1 (3.1)	1 (7.1)	1.00
PCSK9 inhibitor (%)	0 (0.0)	7 (50.0)	<0.001

a. Converted to equivalent dose of atorvastatin. PV, pathologic variant; BMI, body-mass index; MI, myocardial infarction; PCI, percutaneous coronary intervention; HDL, high-density lipoprotein; LDL, low-density lipoprotein; WBC, white blood cell, hs-CRP, high-sensitivity C-reactive protein; HbA1c, glycated hemoglobin; eGFR, estimated glomerular filtration rate; DLCN, Dutch Lipid Clinic Network; ICD, International Classification of Diseases.
